# Phase Field Modelling of Abnormal Grain Growth

**DOI:** 10.3390/ma12244048

**Published:** 2019-12-05

**Authors:** Ying Liu, Matthias Militzer, Michel Perez

**Affiliations:** 1 The Centre for Metallurgical Process Engineering, The University of British Columbia, Vancouver, BC V6T1Z4, Canada; raina.liu.2014@gmail.com; 2 MATEIS, UMR CNRS 5510, INSA Lyon, Univ. Lyon, F69621 Villeurbanne, France; Michel.Perez@insa-lyon.fr

**Keywords:** abnormal grain growth, phase field modelling, high mobility boundaries, disorientation, texture components

## Abstract

Heterogeneous grain structures may develop due to abnormal grain growth during processing of polycrystalline materials ranging from metals and alloys to ceramics. The phenomenon must be controlled in practical applications where typically homogeneous grain structures are desired. Recent advances in experimental and computational techniques have, thus, stimulated the need to revisit the underlying growth mechanisms. Here, phase field modelling is used to systematically evaluate conditions for initiation of abnormal grain growth. Grain boundaries are classified into two classes, i.e., high- and low-mobility boundaries. Three different approaches are considered for having high- and low-mobility boundaries: (i) critical threshold angle of grain boundary disorientation above which boundaries are highly mobile, (ii) two grain types A and B with the A–B boundaries being highly mobile, and (iii) three grain types, A, B and C with the A–B boundaries being fast. For these different scenarios, 2D simulations have been performed to quantify the effect of variations in the mobility ratio, threshold angle and fractions of grain types, respectively, on the potential onset of abnormal grain growth and the degree of heterogeneity in the resulting grain structures. The required mobility ratios to observe abnormal grain growth are quantified as a function of the fraction of high-mobility boundaries. The scenario with three grain types (A, B, C) has been identified as one that promotes strongly irregular abnormal grains including island grains, as observed experimentally.

## 1. Introduction

Abnormal grain growth (AGG) refers to a subset of grains that will grow excessively at the expense of surrounding normal grains leading to an obvious size advantage of the abnormal grains [1,2]. The AGG phenomenon has been observed in many materials, including steels [3,4,5,6,7,8,9,10,11], aluminum alloys [12,13], super alloys [14,15,16], ceramics [17,18,19,20,21], nanocrystalline materials [22,23,24,25] as well as thin films [25,26,27,28]. AGG is of important technological relevance and must be controlled in the thermal processing of polycrystalline materials. It is often undesired since it may lead to heterogeneous microstructures that result in unacceptable material properties. For example, Furnish et al. [23] showed that AGG leads to fatigue failure in nanocrystalline Ni–Fe. In some cases, however, AGG is a useful microstructure engineering concept, also known as secondary recrystallization, e.g., for the development of Goss or cube textures in electrical steels [4,9,10,11,29]. Similarly, AGG is beneficial for magnetostrictive Fe–Ga alloys as well as Fe-based shape memory alloys [30,31]. Further, Kusama et al. [32] demonstrated recently the formation of ultra-large single crystals by AGG. Thus, AGG studies remain an area of active research with an emphasis on electrical steels [9,10,11]. Furthermore, recent investigations deal with AGG during sintering [33,34,35,36] where, however, porosity plays a critical role [37]. Other studies consider the role of local plastic strain on the formation of abnormal grains [24,38,39]. In the context of the present study, AGG will be considered for polycrystalline materials without any external driving pressures or porosity.

The physical mechanisms for AGG may vary between different materials and they are still widely debated. There is, however, a general agreement that for a grain to grow abnormally it must have a persistent growth advantage over its neighbouring grains. The mechanisms of persistent AGG include anisotropy in grain boundary energy (wetting phenomenon), anisotropy in grain boundary mobility, selective unpinning of grain boundaries due to dissolution of precipitates and transitions in grain boundary structures with associated mobility changes [40,41,42,43,44,45]. In essence, all these mechanisms produce highly mobile boundaries that promote AGG. Important characteristics of AGG are complex grain shapes and island grain formation in addition to the continued grain size advantage [46]. The resulting grain size distribution is typically bimodal and can be used to characterize the grain growth process [47].

Recently a variety of computational methods have been used to simulate AGG, such as Monte Carlo (MC) models [2,48,49,50], the vertex model [51,52] and phase field modelling (PFM) [53,54,55,56]. An emphasis of these simulations has been to analyze the role of sub-boundaries, pinning particles and texture on AGG. Suwa et al. [55] included in their 3D-PFM grain growth simulations the role of two texture components, A and B, with the A–B boundary having a five times larger mobility than all other boundaries. They found that when a minority texture component is present, e.g., with a fraction of 0.03 in the initial grain structure, AGG features are predicted due to the rapid growth of these minority grains to comparatively large sizes prior to their impingement. More recently, DeCost and Holm [2] analyzed the phenomenology of AGG using a 2D MC Potts model in a system with three grain types (or three texture components) by simulating a single initially sufficiently large candidate grain of one grain type that is embedded into a matrix of grains which are randomly assigned one of the two other grain types according to a pre-set fraction of each type. The boundary of the candidate grain with grains of one of the other grain types has a mobility advantage of 1000 in these simulations resulting in AGG when sufficient high-mobility boundaries are present. In a few selected cases, as low as 20% of high-mobility boundaries are sufficient for AGG but only when the percolation threshold of 50% is reached do all candidate grains continue to grow abnormally. The initiation stage of AGG is, however, not considered in detail in these simulations.

In this work, PFM is employed for a systematic parametric study to identify mobility advantage conditions that are required for AGG to be initiated. Those mobility advantages are introduced through three different scenarios, which are disorientation angles, two and three grain types, respectively. In the absence of a clear bimodal grain size distribution a grain that is at least five times larger than the average grain size can typically be considered as an abnormal grain.

## 2. Methodology 

During the past few decades, PFM has emerged as a versatile tool to simulate microstructure evolution. PFM has several advantages compared to other computational methods as there is no need to track interfaces explicitly. Therefore, PFM is a powerful methodology to deal with complex morphological features that are often observed for abnormal grains. In addition, phase field models provide outputs in a physical time rather than that of a numerical time as in MC models. In particular, multi-phase field modelling (MPFM) is frequently used as a computational method to simulate the microstructure evolution of metallurgical phenomena, including phase transformation [57,58], recrystallization [59,60] and grain growth [61,62,63]. As a diffuse-interface model, MPFM uses a finite thickness to describe the interfaces, within which the physical properties change continuously. A series of phase field (or order) parameters $\phi_{i}$ is used to describe the microstructure where $\phi_{i}$ equals 1 within grain *i* and its value changes continuously from 1 to 0 across the grain boundary. To identify abnormal grain growth conditions, we employ the commercial software MICRESS where for grain growth simulations the MPFM formulation according to Eiken et al. [64] is used such that the evolution of $\phi_{i}$ for isotropic grain boundary energies, *σ*, is governed by

(1) $$\frac{\partial \phi_{i}}{\partial t}=\overset{N}{\underset{j=1}{\sum }}\frac{\mu_{ij}\sigma_{ij}}{N}\left(\nabla^{2}\phi_{i}-\nabla^{2}\phi_{j}+\frac{\pi^{2}}{\eta^{2}}\left(\phi_{i}-\phi_{j}\right)\right)$$

here $\mu_{ij}$ and $\sigma_{ij}=\sigma$ represent boundary mobility and interfacial energy, respectively, *η* is the grain boundary thickness and *N* is the local number of phase field parameters that are not zero.

**Figure 1:**
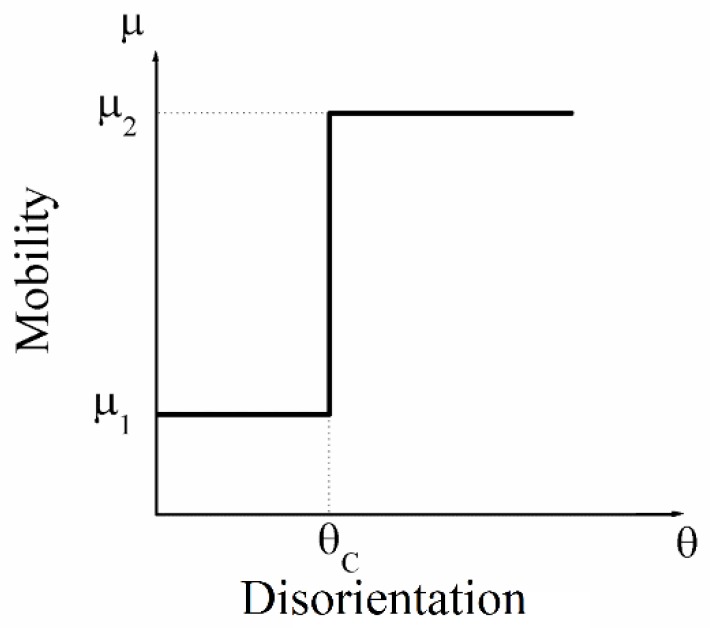
The relation of boundary mobility and disorientation angle.

Boundary mobility advantages are essential for AGG to occur. Here, we consider three different ways to introduce high-mobility boundaries. In the first approach, mobility advantages are defined through a critical disorientation angle. Grains in the initial grain structure are assigned randomly a crystallographic orientation such that each individual boundary is characterized by a disorientation angle *θ*. Then the grain boundary mobility can be formulated as a function of disorientation angle, μ(θ)=f(θ). For the sake of convenience, a simplified model is used as shown in Figure 1. Here, a critical disorientation angle (or threshold angle) $\theta_{C}$ is introduced to select a portion of grain boundaries to be fast boundaries. When the disorientation angle θ is larger than $\theta_{C}$, the boundary mobility is $\mu_{2}$. If the disorientation angle θ is less than $\theta_{C}$, the boundary mobility is $\mu_{1}$. The value of $\mu_{2}/\mu_{1}$ (>1) is the mobility ratio.

Another method to introduce boundary mobility advantages is through different grain types or texture components. Both two and three grain type systems are considered in our simulations. When two grain types A and B are present, there are three types of grain boundaries, i.e., A–A, B–B and A–B boundaries. If one more grain type C is added then there are six types of grain boundaries, i.e., A–A, A–B, A–C, B–B, B–C and C–C boundaries. In both scenarios, the A–B boundaries are selected to be high-mobility boundaries (with mobility $\mu_{2}$) and all the remaining grain boundaries are low-mobility boundaries (with mobility $\mu_{1}$). In the present MICRESS simulations, different grain types (i.e., A, B, C) are approximated as different phases that have the same Gibbs free energy, i.e., the entropy of fusion is infinitively small (10^−7^ J⋅cm^−3^⋅K^−1^) such that there is no additional driving pressure due to the presence of these hypothetical phases.

All simulations of this study are 2D simulations with periodic boundary conditions. For the critical disorientation angle scenario, it is crucial to have a statistically relevant size of the simulation domain. Thus, a square domain with 800 × 800 grid points is used within which 1800 grains are positioned through Voronoi tessellation. Similarly, for simulations with different grain types a domain of 850 × 850 grid points and 1800 grains in the initial structure is used. However, to increase computational efficiency, a smaller domain of 300 × 300 grid points and 120 grains including one candidate grain for abnormal growth in the initial structure is employed for selected simulations with three grain types. For all simulations, the grid spacing is taken to be 1 µm and the high-mobility value $\mu_{2}$ is set to 5 × 10^−2^ cm^4^/(J s). The low-mobility value is then obtained by multiplying the above value with the reciprocal of the selected mobility ratio. For instance, if the mobility ratio is 100, then the mobility is reduced by 0.01 for the low-mobility boundaries.

Sensitivity tests were performed to analyze the role of the selection of numerical parameters, i.e., interface thickness, time step etc. For instance, to test the sensitivity of time step selection, a simulation using automatic time stepping was compared to simulations with constant time steps of 0.002 s and 0.004 s, respectively, while keeping all other settings the same. Similarly, the interface thickness was selected to be 4 and 6 grid points. Based on the sensitivity analysis it was concluded that simulation results are not affected by the selection of these numerical parameters. Thus, all simulations for the parametric study were conducted with automatic time stepping and an interface thickness of 4 grid points to minimize computational cost.

The simulated grain structure evolution is quantified by measuring, at selected times, the area of each grain *i* based on its phase field parameter whereby grid points in the grain boundaries contribute with a fraction that is given by the value of the phase field parameter $\phi_{i}$. From the grain area, the equivalent area diameter (EQAD) of each grain is determined. The mean EQAD is obtained from the mean grain area and the normalized diameter of a grain is introduced as its EQAD divided by the mean EQAD.

## 3. Results

### 3.1. Mobility Advantage through Critical Disorientation Angle

**Figure 2:**
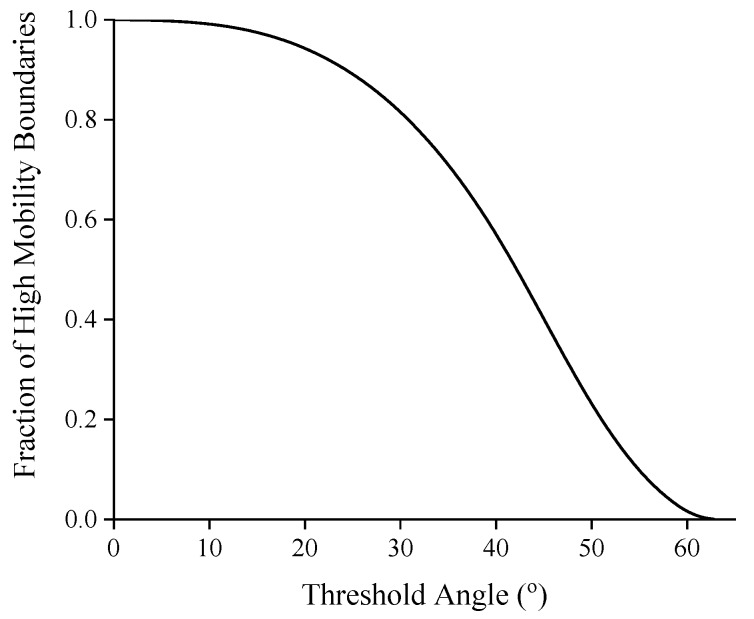
Fraction of high-mobility boundaries as a function of threshold angle in a Mackenzie distribution.

**Figure 3:**
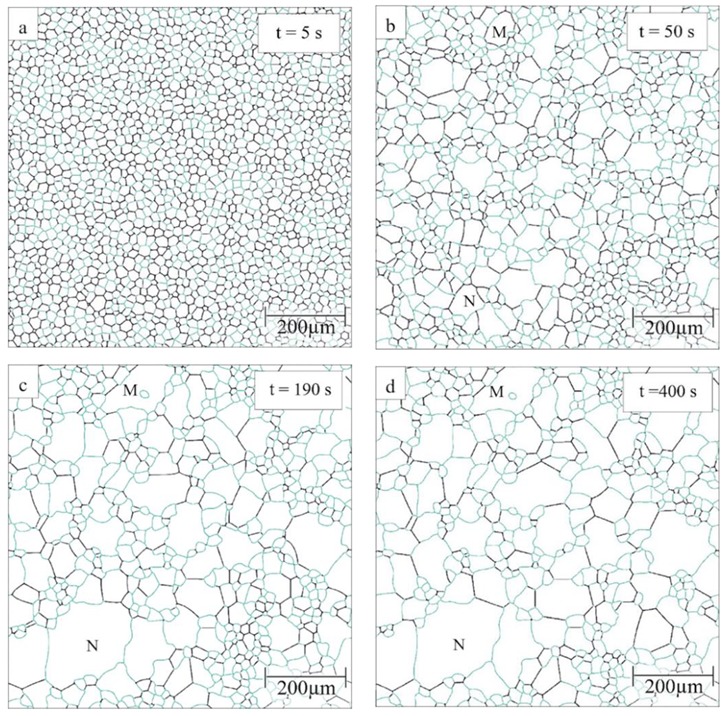
Evolution of grain structure for $\mu_{2}/\mu_{1}=1000$ and $\theta_{C}=40{}^{\circ}$ where high-mobility boundaries are shown in black and low-mobility boundaries in blue: (**a**) t = 5 s, (**b**) t = 50 s, (**c**) t = 190 s, (**d**) t = 400 s. M and N refer to examples of larger grains in the distribution.

The 1800 grains in the initial structure are assigned randomly a cubic crystallographic orientation such that the disorientation distribution follows the Mackenzie distribution [65]. Then the fraction of fast boundaries is a function of the threshold angle, as shown in Figure 2. The potential for AGG will be maximized by having a good mix between fast and slow boundaries, e.g., for threshold angles between 40° and 45° the percentage of highly mobile boundaries falls in the range of 40% to 58%. As an example, Figure 3 shows the evolution of the grain structure with a threshold angle of 40° and a mobility ratio of 1000. Initially all grains are of a similar size as a result of Voronoi tessellation. Gradually the grain size distribution widens and after about 50 s grains M and N are examples of the somewhat larger grains in the distribution. At 190 s, the size advantage of grains M and N has further increased as these grains have comparatively rapidly consumed their much smaller neighbouring grains. In grain M an island grain has formed which is a characteristic sign of AGG. After approximately 400 s, however, grains M and N have, at least to some extent, lost their size advantage and the grain structure starts to approach that of a normal grain size distribution.

**Figure 4:**
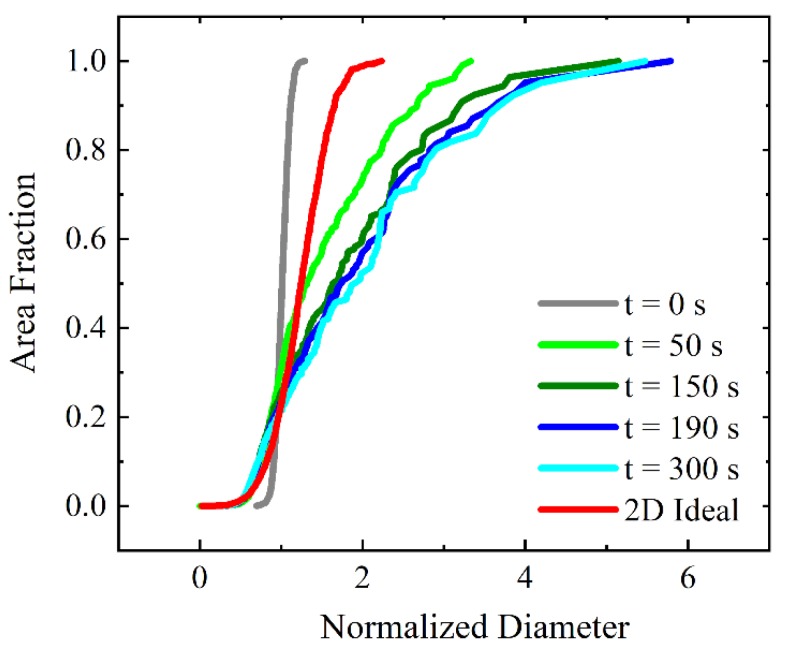
Time evolution of cumulative grain area distribution ($\mu_{2}/\mu_{1}=1000$ and $\theta_{C}=40{}^{\circ}$). For reference, the scaling distribution for 2D ideal grain growth is shown as well.

To more quantitatively analyze the changes in the grain structure with time including AGG stages, the evolution of the cumulative grain area distribution is shown in Figure 4 as a function of the normalized diameter. The red curve provides as reference the cumulative area distribution of normal grain growth that is obtained when all boundaries have the same mobility (mobility ratio of 1). Since normal grain growth is a self-similar process, the red curve represents the resulting scaling distribution where the maximum EQAD is about twice as large as the mean EQAD. The scaling distribution is used as a benchmark to measure the abnormality of grain growth. Initially, the grain area distribution resulting from Voronoi tessellation is much narrower than the scaling distribution but quickly broadens and its width surpasses that of the scaling distribution. After 50 s the maximum EQAD (i.e., that of grain N) is about 3.5 times larger than the mean EQAD. The maximum normalized diameter increases further to 6 at 190 s before it starts to decrease towards a value of 5 for larger times. Thus, there is a particular time (or time period) where the grain area distribution has its broadest range and the maximum normalized diameter obtained for this situation may be taken to assess the abnormality of grain growth for the selected grain growth parameters, i.e., threshold angle and mobility ratio. Therefore, one can find the widest distribution curve for each scenario to quantify the degree of AGG.

**Figure 5:**
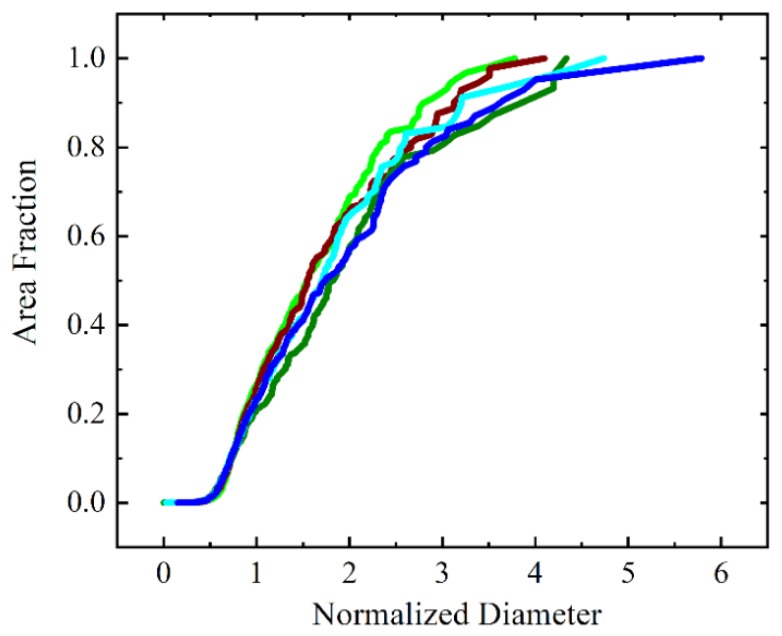
Widest cumulative grain area distribution for five different initial structures with $\theta_{C}=40{}^{\circ}$ and $\mu_{2}/\mu_{1}=1000.$

The scenario described above does show some indication of AGG that is based on the presence of one or a few fast-growing grains. It is thus important to re-evaluate AGG events starting from different initial structures. The widest distribution curves resulting from a range of initial structures are shown in Figure 5 where the maximum EQAD is varying from 3.5 to 6. With the same amount of highly mobile boundaries, their relative positions significantly affect the extent of AGG. The highest value for the maximum EQAD has indeed been observed for the case shown in Figure 3 and Figure 4 and this has also been the only case with the formation of an island grain illustrating that AGG is a rather rare event for the present threshold angle scenario. For a grain to grow abnormally, a sustained mobility advantage is required and for the investigated threshold angle case the required mobility advantage can only be attained for very few grains and a limited time period.

**Figure 6:**
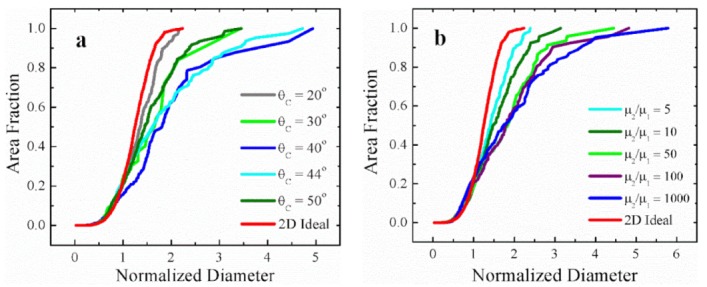
Cumulative grain area distribution for the widest grain size distributions: (**a**) effect of threshold angle when $\mu_{2}/\mu_{1}=100$; (**b**) effect of mobility ratio when $\theta_{C}=40{}^{\circ}.$ For reference, the scaling distribution for 2D ideal grain growth is shown as well.

To quantify the role of threshold angle and mobility ratio on AGG, we select the initial structure of the above case with the most pronounced AGG structure (see Figure 3) for a parametric study. The widest distribution curves of each scenario are presented in Figure 6 to show the effect of threshold angle and mobility ratio, respectively, on growth abnormality. When changing the threshold angle from 20° to 50°, it is confirmed that growth abnormality is restricted to the threshold angle range of 40–44° where the percentage of the fast boundaries is around 50% (i.e., here 58% and 45%, respectively). For threshold angles of 30° and 50°, on the other hand, 82% and 22% of the boundaries are highly mobile, essentially eliminating the conditions for individual grains to have a sufficient growth advantage and restricting the maximum normalized diameter to about 3, i.e., a grain structure that approaches that of normal grain growth. Similarly, when changing the mobility ratio from 5 to 1000 for a threshold angle of 40°, one can conclude from Figure 6b that when the mobility ratio is 10 or less, grain growth does not occur abnormally since the distribution curves are sufficiently close to that for normal grain growth. Mobility ratios above 50 lead to AGG where the severity of AGG is augmented when increasing the mobility ratio from 100 to 1000 as the associated maximum normalized diameter increases from 5 to 6.

**Figure 7:**
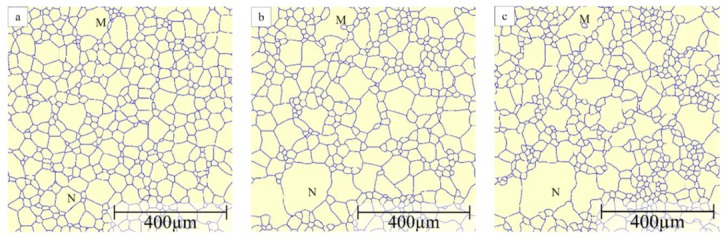
Grain structure for the widest grain size distribution when $\theta_{C}=40{}^{\circ}$ and (**a**) $\mu_{2}/\mu_{1}=10$; (**b**) $\mu_{2}/\mu_{1}=100$; (**c**) $\mu_{2}/\mu_{1}=1000$.

The influence of mobility ratio is further illustrated in Figure 7 by comparing the grain structure images with the widest size distribution. For a mobility ratio of 10, there are no obvious grain size advantages and no tendency of island grain formation. However, if the mobility ratio is increased to 100, grain N has a clear size advantage over the other grains and the size advantage of grain N becomes even more pronounced when the mobility ratio is further increased to 1000. Meanwhile, with a mobility ratio of 100, grain M shows a tendency to embrace one of its small neighbour grains but an island grain has not yet formed. If the mobility ratio is further increased to 1000, there is an island grain formed within grain M.

Whilst in these simulations with a random distribution of the highly mobile boundaries the potential of at least mild AGG is confirmed, it is a rather rare event. Extensive AGG appears to be limited as the very few grains that acquire temporarily an appreciable size advantage stop to grow rapidly as soon as they attain a situation where they are entirely surrounded by low-mobility boundaries. 

### 3.2. Mobility Advantage in Two-Grain Type Systems

**Figure 8:**
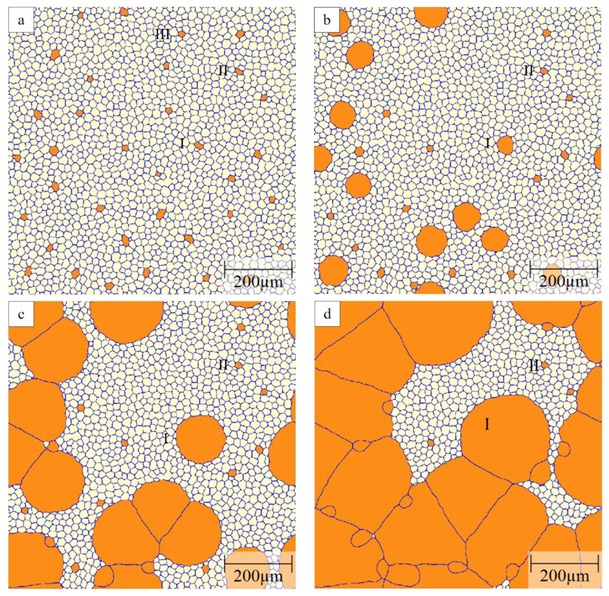
Microstructure evolution of A–B system with fast A–B boundaries when $\mu_{2}/\mu_{1}=1000$ and 0.67% growing B grains in the initial structure: (**a**) t = 0 s; (**b**) t = 25 s; (**c**) t =50 s; (**d**) t = 75 s. B grains shown in orange and A grains in white.

Another method to introduce a mobility advantage is through different grain types or texture components. Here, we start with a two-component system consisting of A and B grains where the A–B boundaries are taken as highly mobile and their fraction is then determined by the fraction of B grains in the initial microstructure. An example of evolution of the grain structure in the A–B system is shown in Figure 8 for a mobility ratio of 1000 and 2% of B grains randomly distributed in the initial structure with a narrow size distribution resulting from Voronoi tessellation. Because of the small fraction of B grains, all of these B grains have initially only A grains as their neighbours and are, thus, surrounded entirely by highly mobile grain boundaries. As a result, there is a rapid evolution of the B grains. Whether or not they grow or shrink depends on their size with respect to their neighbours, i.e., smaller B grains will shrink and larger B grains will grow. After a short time (t = 25 s), about 1/3 of the B grains (e.g., grain III) have shrunk and disappeared very quickly in the scenario shown in Figure 8. Approximately 1/3 of B grains (e.g., grain I) grow rapidly at the expense of the surrounding A grains, thereby forming a bimodal grain size distribution that is characteristic for abnormal growth. The remaining 1/3 of B grains with a hexagonal structure (e.g., grain II) form, at least for some time, a stable grain structure with their neighbours that is a specific feature of 2D grain growth which does not exist in 3D. The maximum normalized diameter depends, then, primarily on the spacing of the growing B grains which, in the present case, constitute 0.67% of all grains in the initial structure. Impingement of growing grains occurs later, see e.g., images at 50 and 75 s in Figure 8.

**Figure 9:**
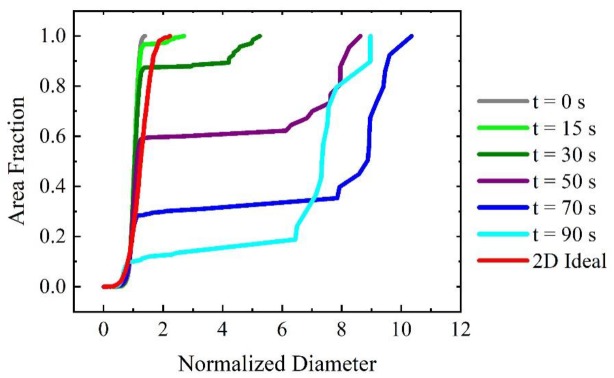
Time evolution of cumulative grain area distribution in the A–B system ($\mu_{2}/\mu_{1}=1000,$ growing B = 0.67%). For reference, the scaling distribution for 2D ideal grain growth is shown as well.

As discussed above for the threshold angle method, one can plot the evolution of the cumulative grain area distribution as a function of normalized diameter to further evaluate the abnormality of grain growth, as presented in Figure 9. The red curve is the scaling distribution of normal grain growth as a reference. The initial grain size distribution resulting from Voronoi tessellation is very narrow before a bimodal distribution emerges quickly due to the rapid growth of a few of the B grains. The bimodality of the distribution is represented by the plateau in the cumulative grain area distribution, i.e., there are two populations of grains in the structure consisting of small grains (here the A grains and B grains that do not grow) and large grains (i.e., the growing B grains) with virtually no size overlap. With time the size of the growing B grains and their area fraction rapidly increases. For example, after 30 s the maximum normalized diameter is 5 and the area fraction of the large grains is about 0.1. After 70 s the largest normalized diameter of about 11 is reached and the area fraction of the large grains is increased to about 0.7. Eventually, the growing B grains impinge (see Figure 8) and have consumed all A grains. As a result, the overall grain structure approaches that of a normal grain size distribution of the coarse B grains and the maximum normalized diameter starts to decrease at longer times. As a specific feature of the 2D grain growth simulations, the initially stable hexagonal B grains are incorporated into the B grain microstructure as shown in Figure 8d. These smaller B grains remain almost frozen for some time as their shrinkage requires migration of the low-mobility B–B grain boundaries thereby retaining technically a bimodal structure for some time with, however, an increasingly marginal area fraction of the small grains, e.g., after 90 s the area fraction of the small grains is reduced to about 0.1 for the case shown in Figure 9. The residual bimodality may, however, be considered as an artefact of 2D simulations.

Even though a clearly bimodal grain size distribution can be obtained with the above mobility scenario, it does not show the development of any irregular grains with complex morphologies that are frequently observed in AGG. The growing B grains show a circular evolution in the 2D simulations, which would translate into spherical growth in 3D, i.e., the grain structure while consisting of two grain types with different sizes remains morphologically equiaxed.

**Figure 10:**
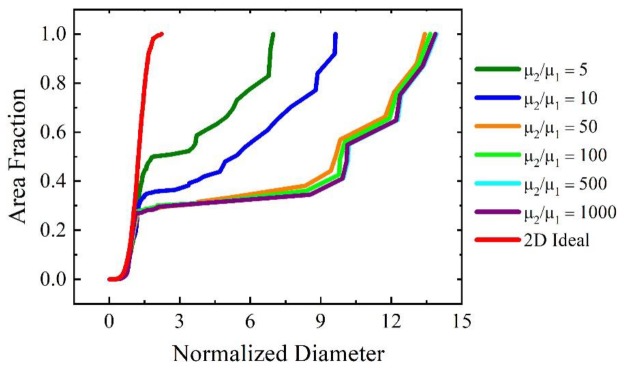
Effect of mobility ratio on cumulative grain area distribution for widest grain size distributions in the A–B system with 0.44% growing B grains in the initial structure. For reference, the scaling distribution for 2D ideal grain growth is shown as well.

Even so, similarly to the threshold angle approach we performed a systematic parameter study to explore the influence of mobility ratio and initial percentage of growing B grains on the extent of the bimodality of the grain structure. Taking the scenario with 0.44% of growing B grains in the initial structure the mobility ratio is varied from 5 to 1000 and the widest distribution curves are summarized in Figure 10. For mobility ratios larger than 50, the distribution curves are very close to each other with a maximum normalized diameter of about 14, i.e., the degree of abnormality is not sensitive to mobility ratios larger than 50. For a mobility ratio of 5 and 10 the bimodality is less severe with a maximum normalized diameter of 7 and 9, respectively. Changing the percentage of growing B grains for a given mobility ratio (here 1000) affects the average distance between these grains and thus the maximum normalized diameter that can be attained decreases from 14 for 0.44% of the initial growing B grains to about 5 when 3.33% of B grains in the initial structure grow. The latter may be taken as threshold for AGG.

### 3.3. Mobility Advantage in Three-Grain Type Systems

In the two-grain component systems, most (and for sufficiently small fractions all) growing B grains are entirely surrounded by highly mobile A–B grain boundaries and grow rapidly in a regular, equiaxed fashion. In contrast, much more irregular shapes of abnormal grains are observed in many experimental AGG scenarios, including for 2D grain growth. To add complexity into our simulations, a third grain type C is introduced as a result of which growing B grains will have a mixture of fast A–B boundaries and slow B–C boundaries. The grain types are randomly assigned in the initial structure to match a pre-scribed fraction of each component.

**Figure 11:**
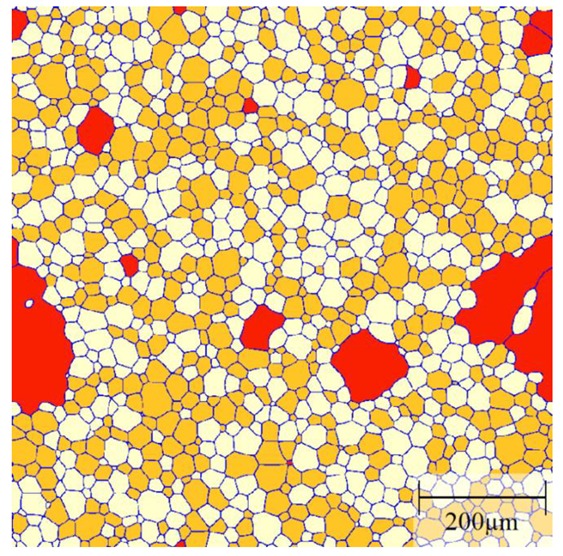
Typical grain structure in a three-grain type system (initial composition of B% = 1%, A% = C% = 49.5%) with a mobility ratio of 1000. Grains of type A, B and C are shown in orange, red and white, respectively.

Figure 11 shows a typical example of the grain structure obtained in the three-grain type simulations with a mobility ratio of 1000. Here, the red grains represent the B grains; and orange and white grains are defined as A and C grains, respectively. Initially all grains have a similar size and 1% B grains are randomly distributed in the A–C grain matrix with equal fractions of A and C grains. Similar to the two-grain type systems, some B grains with a smaller size will shrink and disappear quickly. The second group of B grains with a hexagonal structure will neither shrink nor grow and only those B grains with a size advantage over their neighbours will grow. A few of these growing B grains show clear signs of abnormal grains, i.e., complex grain morphologies including the formation of island grains.

**Figure 12:**
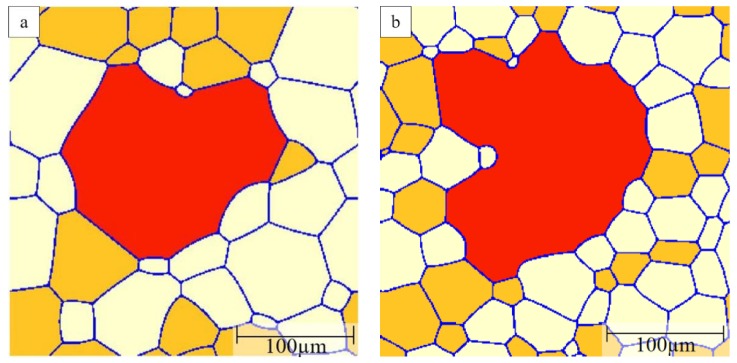

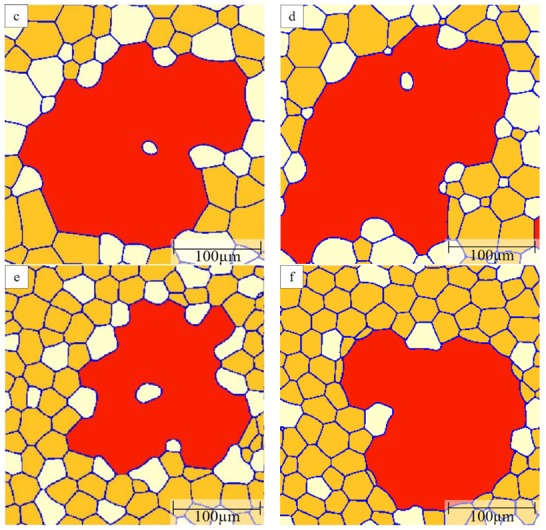
Structure of rapidly growing B grain (shown in red) in three grain type systems with a mobility ratio of 1000 when (**a**) A = 40%; (**b**) A = 50%; (**c**) A = 60%; (**d**) A = 70%; (**e**) A = 80%, (**f**) A = 90% where A grains are in orange and C grains in white.

For computational efficiency, subsequent simulations were performed in a smaller domain to focus on the behavior of one candidate-growing B grain. The percentage of A grains will influence the fraction of the highly mobile A–B boundaries that will change with time depending on the local environment of the growing B grain. Figure 12 illustrates examples of the obtained grain morphologies of the B grain for different A grain percentages in the initial microstructure and a mobility ratio of 1000. When there are 40% A grains in the matrix, the B grain grows in a regular, equiaxed fashion with a polygonal shape that is more realistic than the circular shapes in the two texture component systems (Figure 12a). Because some of the grain boundaries are fast A–B boundaries the B grain becomes the largest grain in the structure but its size advantage is rather modest such that the overall grain structure appearance is close to that for normal grain growth. Increasing the percentage of A grains leads to a higher fraction of fast A–B boundaries, thereby promoting the growth of the B grain into an abnormally large grain. For an A grain percentage of 50%, the B grain evolves into an abnormally large grain with a more complex shape (Figure 12b). Increasing the A grain percentages to 60%, 70% and 80%, the B grain becomes an even more prominent abnormal grain that also includes island grains that belong to grain type C (Figure 12c–e). These island grains form when the growing B grain locally reaches a situation where A grains completely surround a C grain. As the B grain can consume these A grains rapidly an island grain will result within the B grain. These island grains are unstable but will remain for some time as they can only be eliminated by migration of the low-mobility B–C boundaries. The probability of the island formation hinges on a combination of a sufficiently high percentage of A grains with a still sizeable percentage of C grains. Increasing the A grain percentage to 90%, the B grain approaches a more equiaxed, circular shape but with some local inlet type features due to pinning by some small C grains (Figure 12f). When increasing the A percentage further, the evolution of the B grain becomes increasingly similar to the situation of the two-grain type system discussed above in Section 3.2.

**Figure 13:**
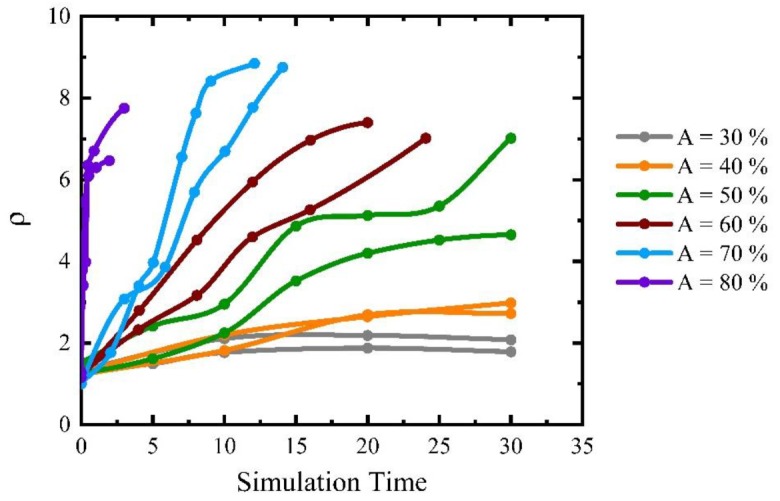
Effect of percentage of A grains on the size evolution of grain B for a mobility ratio of 1000. The size of grain B is shown in units of the average grain diameter of A and C grains.

The details of the evolution of grain B depend also on the initial grain structure. Figure 13 compares two runs from different initial structures with otherwise unchanged parameters in terms of A percentage and mobility ratio (here 1000). The evolution of grain B is shown as its normalized grain diameter defined by:

(2) $$\rho =\frac{d_{B}}{d_{AC}}$$

where $d_{B}$ is the EQAD of the B grain and $d_{AC}$ represents the average EQAD of the matrix grains consisting of A and C grains. As expected, there is some variation in detail due to the initial structure but the general conclusions are less affected. The percolation threshold of 50% A grains is required to consistently reach the 5 grain diameter threshold for AGG. The B growth rate increases with the A grain percentage and the associated increase of fast A–B boundaries and the maximum size of B grains (here about 9) is found for A grain percentages of 70% and higher. It must be noted, however, that the size advantages are here limited by the smaller domain size of the simulations. As discussed for the A–B scenario, the size advantage is also critically dependent on the spacing between abnormally growing grains.

In addition to size advantage, complex grain shapes are an important characteristic of AGG. To quantify the grain morphology, the circularity, ε, defined by

(3) $$\varepsilon =\frac{4\pi A}{P^{2}}$$

may be used where *A* is the area of the B grain and *P* its perimeter. In the most pronounced AGG cases, i.e., for 60%, 70% and 80% A, the circularities of the fully developed abnormal B grains fall into the 0.45–0.70 range.

For cases with comparable grain size the number of grain neighbours is an alternative way to evaluate the morphological complexity. Taking the examples of Figure 12, the number of B grain neighbours increases from 13 for 40% A to 22 for 50% A, 25 for 60% A and 33 for 70% A but then decreases to 26 and 27 for 80% and 90% A, respectively. The larger grain number of neighbours for 70% A is consistent with the more complex grain morphology in this case that includes an island grain. Interestingly, the majority of the neighbours are small C grains that form low-mobility B–C boundaries such that these grains are obstacles for the progression of the faster moving A–B boundaries leading to the formation of peninsula-type grains or even island grains. All these abnormally large grains have still a fraction of highly mobile boundaries that increases from about 1/3 for 40%, 50% and 60% A to 0.4 for 70% A, 0.6 for 80% A and 0.8 for 90% A.

The above simulations in the three-grain type system have been carried out with a mobility ratio of 1000. To identify regions of AGG it is also of interest to consider the role of mobility ratio in addition to A percentage that provides a measure of the fraction of highly mobile A–B boundaries. Taking the 5 grain diameter size advantage as a threshold a mobility ratio of 30 is required for 50% A whereas mobility ratios of 10 and 5, respectively, are sufficient for 70% and 100% A, respectively.

### 3.4. Comparison with Monte Carlo Simulation

**Figure 14:**
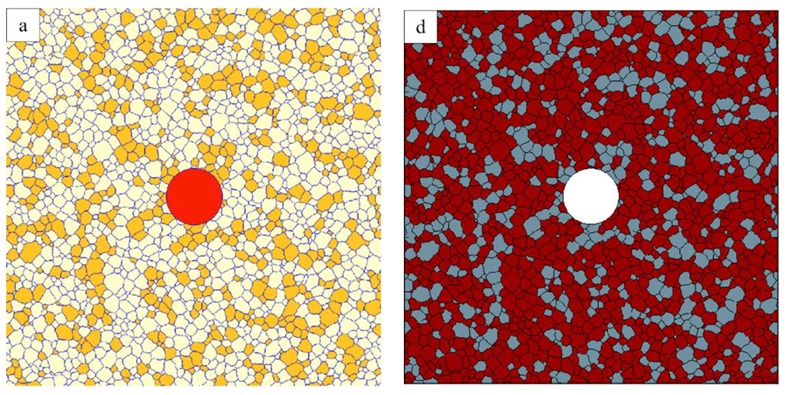

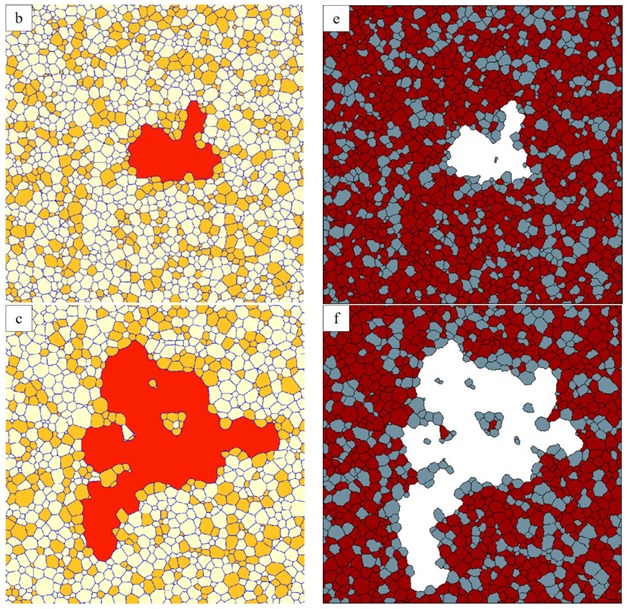
Comparison between phase field modelling (PFM, left) and Monte Carlo (MC, right) [2] simulations with the same initial microstructure: (**a**) initial structure for PFM, (**b**) initial structure for MC, (**c**) intermediate structure for PFM, (**d**) intermediate structure for MC, (**e**) final structure for PFM, (**f**) final structure for MC. The B grain is shown in red for PFM and in white for MC simulations. The A grains are in white (PFM) and dark red (MC) and the C grains are in orange (PFM) and grey (MC), respectively.

PFM and MC simulations are effective and versatile methods to study AGG. However, whether the simulation methods will influence the simulation results is of great interest. Figure 14 compares the results of PFM and MC simulations. The MC simulation results are taken from DeCost and Holm [2]. Both simulations start from the same initial structure with 68% A grains, as shown in Figure 14a,d, and the mobility ratio is set to be 1000. In PFM, the B grain is in red while A and C grains are shown in white and orange, respectively. In the MC method [2], the white grain represents the B grain; red and blue grains are defined as A and C grains, respectively. Comparing Figure 14b,e, a small island grain is found in Figure 14e [2] while at the same position, this small C grain has already been consumed in the PFM simulation. After running the simulation for a longer time, some island grains also start to form in the PFM simulation, as shown in Figure 14c. However, in the MC model, there are many more island grains found as presented in Figure 14f. [2] The difference may be caused by the straightening effect of grain boundaries in PFM. In PFM, in order to reduce the total free energy, the grain boundaries tend to be straight lines thus losing the driving pressure due to curvature. Meanwhile, in the MC simulation, there is no such straightening effect. Nevertheless, apart from these minor differences, the overall coarsening mode of the abnormal grain is in both simulations very close to each other.

Since the time scales in PFM and MC simulations are different, it is of interest to match the two time scales and develop a more quantitative way to analyze the consistency of the two computational methods. In PFM, the simulation time is the real phase field time (PFT), and the unit is second. In the MC simulation, the time scale is measured by the Monte Carlo step (MCS). To match the two time scales, a constant parameter κ is introduced, where:

(4) t(MCS)=κ·t(PFT)

**Figure 15:**
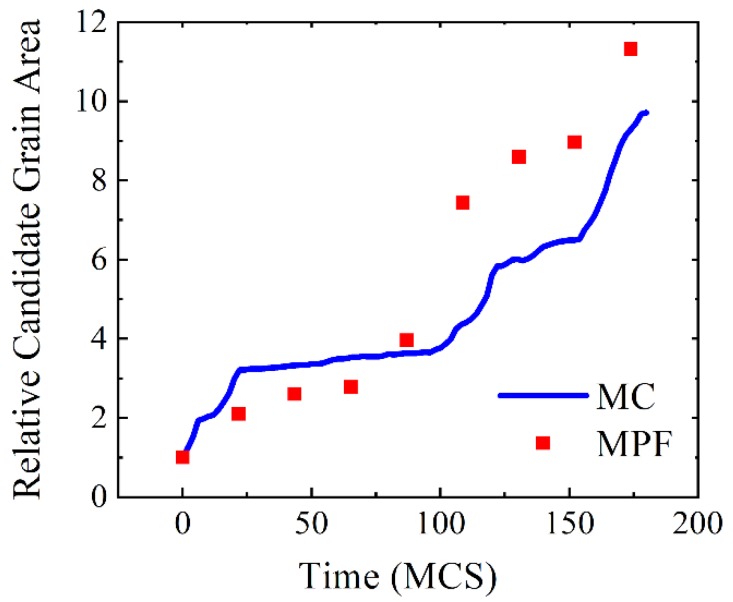
Size evolution of the abnormal grain obtained in PFM and MC simulations in units of the initial grain area of the candidate grain.

Here κ=5.1 is obtained to best match the two simulation results in terms of the size evolution of the abnormal grain. The increase of the candidate grain area is plotted in Figure 15 as a function of simulation time. From this graph, despite of little fluctuations, the overall grain growth paths are almost the same in both simulations.

## 4. Discussion

**Figure 16:**
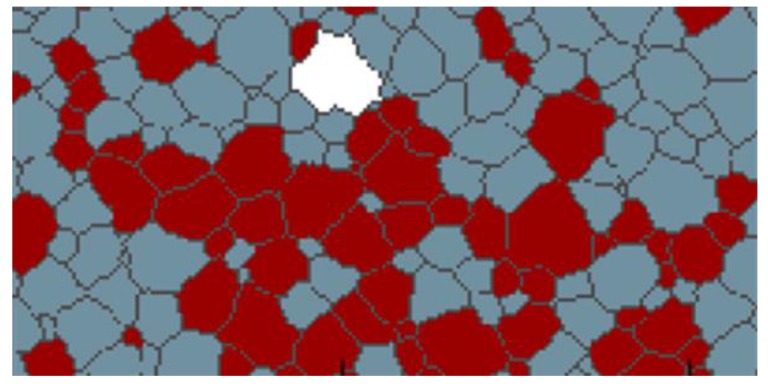
Initial structure for initiation of abnormal growth of a white candidate grain with a fraction of 0.3 for A (red) grains that provide the highly mobile boundaries with the white grain; bottom half of Figure 8a in ref. [2].

Even though there is remarkable agreement between PFM and MC simulations in the above benchmark case, there are in detail some apparently different conclusions that may be drawn from the present PFM simulations as compared to the MC study of DeCost and Holm [2]. For example, they present also a case of initiation of abnormal growth for a situation with 30% A grains (red grains in their paper). Figure 16 shows the local starting environment for this grain indicating that there is a clear percolation path due to the arrangement of A grains such that the white grain can grow into an abnormal grain. Furthermore, when considering growth of an initially large circular candidate grain (see e.g., Figure 14b) they performed 120 independent simulations for each A (red) grain fraction scenario. For 50% A grains, the candidate grain grows in all cases abnormally, for 40% A grains in the majority of the cases abnormal growth occurs whereas for 30% A abnormal growth is recorded in about half of the cases. This provides further evidence that the local A grain environment is critical rather than the global A grain fraction. Furthermore, the initial size advantage promotes the chances for abnormal growth due to the larger numbers of grain neighbours including those providing high-mobility boundaries.

**Figure 17:**
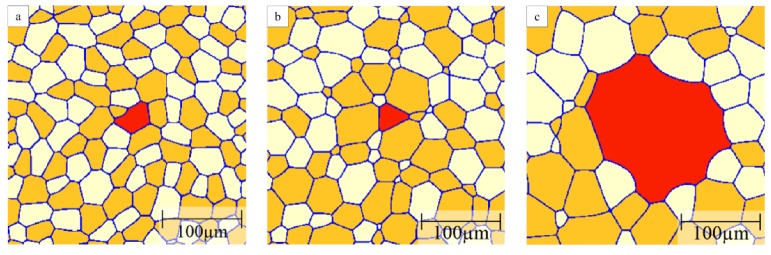
Effect of mobility ratio on growth behavior of candidate B grain (shown in red) for 50% A grains (shown in orange): (**a**) initial grain structure, (**b**) grain structure after 10 s for a mobility ratio of 5, (**c**) grain structure after 30 s for a mobility ratio of 10.

In the present PFM study the focus has been, in addition to the fraction of highly mobile boundaries, to also assess the role of the mobility ratio on the initiation of abnormal growth. Starting from an initial grain structure obtained by Voronoi tessellation the eventually abnormally growing grains had initially only a marginal size advantage (usually with a normalized grain diameter of 1.5 or less). Thus, the growth potential of these grains is limited compared to the cases considered by DeCost and Holm [2] such that the percolation limit is more restrictively enforced. Furthermore, it is not only the local grain environment but also the mobility ratio that determines whether the candidate grain will grow abnormally or even shrink, as illustrated in Figure 17. Here, a scenario is shown where the candidate grain shrinks for a mobility ratio of 5 and will eventually disappear as its A grain neighbours have sufficiently grown (see Figure 17b) whereas for a mobility ratio of 10 the candidate grain develops into an abnormally large grain (see Figure 17c).

## 5. Conclusions

The onset conditions for abnormal grain growth (AGG) have been quantified for the first time with systematic phase field simulations. For AGG to be initiated, selected individual grains must have some grain boundaries with a sufficient mobility advantage compared to other boundaries. Two-dimensional MPFM simulations have been conducted using different ways to introduce highly mobile grain boundaries, i.e., a critical disorientation angle above which the boundaries are highly mobile as well as systems with two (A–B) and three (A–B–C) grain types where the A–B boundaries are assumed to be highly mobile. Systematic parametric studies have been performed for these systems by changing the fraction and distribution of the highly mobile boundaries and the mobility ratio between high and low-mobility boundaries to identify conditions for AGG. The high-mobility boundaries are randomly distributed in the disorientation angle approach and modest AGG scenarios with maximum grain sizes of 5–6 times larger than the mean grain size are obtained for a narrow range of threshold angles where about 40%–60% of the boundaries are highly mobile with mobility ratios of at least 100. AGG with a well-developed bimodal grain structure can readily occur for mobility ratios as low as 5 in the two-grain type system when the fraction of growing B grains with high-mobility boundaries is sufficiently small. The maximum grain size depends primarily on the distance between the growing grains. In the present simulations, a maximum grain size of 14 times larger than the apparent mean grain size has been obtained when 0.4% of the initial grains can grow abnormally. The two-grain type simulations lead, however, to rather unrealistic circular shapes of the abnormally large grains. This aspect can be mitigated when introducing a third grain type in the system where the growing grains are not completely surrounded by highly mobile boundaries. Realistic shapes of abnormally growing grains can be obtained including the formation of island grains for a range of conditions with a sufficiently high fraction of highly mobile boundaries while maintaining a critical amount of the third grain type. Furthermore, the PFM simulations leading to truly abnormal grains are quantitatively similar to MC simulation results by DeCost and Holm [2], as verified with a benchmark simulation.

To establish in more detail AGG conditions, it may be useful to apply machine-learning techniques to the analysis of simulation data bases for the three-grain type systems. So far, all the simulations have been conducted in 2D and provide an important insight into 2D abnormal grain growth observed e.g., in thin films [25,26,27,28]. The simulation results also provide guidance for future 3D simulations to specify in more detail AGG conditions for bulk materials. Furthermore, the above studies apply to pure systems but can be further extended to include the role of precipitate pinning as well as solute drag, which will add complexity to the analysis.
